# Visceral and Subcutaneous Abdominal Fat Predict Brain Volume Loss at Midlife in 10,001 Individuals

**DOI:** 10.14336/AD.2023.0820

**Published:** 2024-08-01

**Authors:** Cyrus A Raji, Somayeh Meysami, Sam Hashemi, Saurabh Garg, Nasrin Akbari, Ahmed Gouda, Yosef Gavriel Chodakiewitz, Thanh Duc Nguyen, Kellyann Niotis, David A Merrill, Rajpaul Attariwala

**Affiliations:** ^1^Mallinckrodt Institute of Radiology, Neuroradiology Division, Washington University in St. Louis, MO, USA.; ^2^Pacific Brain Health Center, Pacific Neuroscience Institute and Foundation, Santa Monica, CA, USA.; ^3^Saint John's Cancer Institute, Providence Saint John's Health Center, Santa Monica, CA, USA.; ^4^Prenuvo, Vancouver, Canada.; ^5^Voxelwise Imaging Technology, Vancouver, Canada.; ^6^Early Medical, Boca Raton, FL, USA.; ^7^Institute of Neurodegenerative Diseases-Parkinson's & Alzheimer's Research Education Foundation, Boca Raton, FL, USA.; ^8^Providence Saint John’s Health Center, Santa Monica, CA, USA.; ^9^Department of Psychiatry and Biobehavioral Sciences, David Geffen School of Medicine, University of California Los Angeles, Los Angeles, CA, USA.; ^10^AIM Medical Imaging, Vancouver, Canada.

**Keywords:** visceral fat, subcutaneous fat, brain volume loss, deep learning

## Abstract

Abdominal fat is increasingly linked to brain health. A total of 10,001 healthy participants were scanned on 1.5T MRI with a short whole-body MR imaging protocol. Deep learning with FastSurfer segmented 96 brain regions. Separate models segmented visceral and subcutaneous abdominal fat. Regression analyses of abdominal fat types and normalized brain volumes were evaluated, controlling for age and sex. Logistic regression models determined the risk of brain total gray and white matter volume loss from the highest quartile of visceral fat and lowest quartile of these brain volumes. This cohort had an average age of 52.9 ± 13.1 years with 52.8% men and 47.2% women. Segmented visceral abdominal fat predicted lower volumes in multiple regions including: total gray matter volume (r = -.44, p<.001), total white matter volume (r =-.41, p<.001), hippocampus (r = -.39, p< .001), frontal cortex (r = -.42, p<.001), temporal lobes (r = -.44, p<.001), parietal lobes (r = -.39, p<.001), occipital lobes (r =-.37, p<.001). Women showed lower brain volumes than men related to increased visceral fat. Visceral fat predicted increased risk for lower total gray matter (age 20-39: OR = 5.9; age 40-59, OR = 5.4; 60-80, OR = 5.1) and low white matter volume: (age 20-39: OR = 3.78; age 40-59, OR = 4.4; 60-80, OR = 5.1). Higher subcutaneous fat is related to brain volume loss. Elevated visceral and subcutaneous fat predicted lower brain volumes and may represent novel modifiable factors in determining brain health.

## INTRODUCTION

North American populations suffer a high prevalence of obesity with approximately 36% in the U.S. and 25% in Canada estimated as obese [1, 2]. Overweight and obese persons combined reach rates of over 60% in each country [1, 3]. High body adiposity across overweight and obese status has numerous cardiovascular risks. These include hypertension, hyperlipidemia, type 2 diabetes mellitus and atherosclerotic heart disease [3, 4]. Such complications, in turn, increase both morbidity and mortality particularly with aging populations. Thus, the elevated cardiovascular risk profile associated with obesity can also influence public health as mitigation and prevention strategies may reduce the rates of subsequent vascular disease.

Multiple prior investigations have suggested a connection between body fat accumulation and increased Alzheimer's dementia (AD) risk. A study by Whitmer et al. (2008) demonstrated that midlife obesity translated to an increased risk of late life AD, suggesting a long-term influence of obesity on brain health [5]. Another study [6] reported that midlife overweight or obese status raised dementia risk with odds ratios of 1.71 and 3.88 respectively. A meta-analysis by Pedditizi et al. (2016), further noted that while midlife obesity does increase dementia risk, the relationship reverses in late life [7]. These results suggest that characterizing obesity earlier in life is important for understanding increased AD risk. Kivimäki et al. confirmed these results in a larger follow up study of 1.3 million persons that showed the risk of dementia in relation to Body Mass Index (BMI) increases when that metric is evaluated greater than 20 years prior to dementia diagnosis versus 10-20 or 10 years [8]. Thus, the public health implications of higher body fat tissue extend beyond cardiovascular considerations and into brain health.

One underlying observation that is thought to explain the high risk of AD with obesity is the increased burden of brain atrophy in this population. An early study demonstrated lower volume as a function of higher BMI in 94 cognitively normal persons who remain so 5 years after their scan [9]. These observations were replicated both in larger community and referral clinic cohort samples [10, 11]. Population based cross-sectional work done with the U.K. Biobank of over 12,000 individuals also showed lower brain volumes related to higher BMI [12].

However, when trying to understand the relationship between body fat tissue, brain health, and potential downstream effects such as higher AD risk, BMI has several limitations. Computed in part by the ratio of weight in pounds to height in inches, BMI is a surrogate of human body fat, not a direct measure, as it includes bone and muscle mass [13]. As such, this measure does not singularly track nor characterize the anatomical distribution of body fat. This distribution is characterized in two main types: visceral fat (vfat) or visceral adipose tissue that deposits around organs and subcutaneous fat (sfat) or subcutaneous adipose tissue, the latter type accounting for 80-90% of variance in BMI while visceral fat only accounts for 10-20% [14]. This distinction has potential importance to brain health as visceral fat has been related to both higher dementia risk [15] and brain atrophy [16]. However, studies examining the relationship between both of these fat types and underlying brain structure in larger populations are lacking in the literature.

We therefore aimed to investigate the associational relationships between visceral, subcutaneous fat and brain structure on MR imaging in a large cross-sectional sample of individuals across the lifespan. Our hypothesis is that visceral fat will be related to lower brain volumes, a biomarker for neurodegeneration [17], from the macrostructural tissue class perspective to lobar brain volumes and Alzheimer disease specific regions affected early in the pathological process: the hippocampus, posterior cingulate, and precuneus. We also evaluated these questions with subcutaneous fat.

## MATERIALS AND METHODS

### Participant Whole Body MR Imaging

All analyses were done with IRB approval (Advarra, WPBP-001). Participants were scanned on 1.5T Philips Ingenia Ambition, Siemens Espree and Aera scanners at the following locations: Vancouver, BC, Canada; Redwood City, CA; Los Angeles, CA; Minneapolis, MN; Boca Raton, FL; Dallas, TX. Each participant received a non-contrast whole body MRI scan that has been previously detailed [18]. Briefly, each scan included whole body coronal T1-and axial T2-weighted with whole body coronal STIR, axial T1 in and out of phase images with Dixon technique [19, 20] allowing for visual identification and quantification of vfat and sfat. Additional whole-body sequences included diffusion weighted imaging (DWI), STIR, and axial T2 BLADE of the chest, abdomen and pelvis. Brain sequences included sagittal 3D T1 MPRAGE, axial 2D FLAIR, and time-of-flight MRA but only 3D T1 MPRAGE brain images were the focus of neuroimaging analysis in this work.

#### Deep Learning Analyses and Quantification of Body Fat

We used a T1 weighted MRI scan of the whole-body to segment the visceral fat among 28 anatomical structures. The dataset consists of 102 Siemens MRI scans, and it was divided into 72 scans for training, 18 for validation, and 12 for testing with an age range of 27-66 years. All anatomical structures were manually annotated by radiologists using ITK-SNAP[21]. QC was performed by another trained radiologist for generating the ground-truth masks. We used nnU-Net model[22], as a fully supervised segmentation architecture for training and inferring the different segmentation classes. The average testing dice score for the visceral fat is (0.8402 ± 0.07). Finally, the visceral fat and subcutaneous fat volumes for each patient were computed in milliliters (ml) by multiplying the number of its predicted voxels by the 3D MRI voxel space.

**Figure 1.**
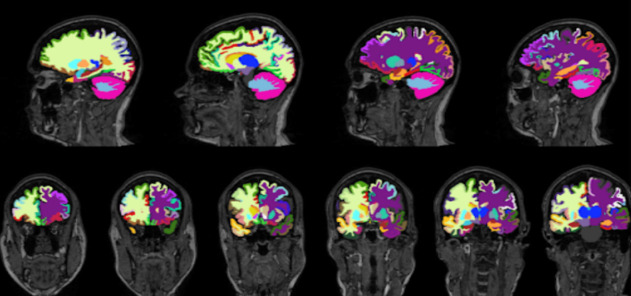
**Brain Volume Segmentation Results**. An example of the FastSurfer segmentation model. The various colors denote different regional segmentations in the brain on a T1 weighted image.

### MRI Volumetric Measurement of Regional Brain Volumes

To generate the respective regional volumetric of the brain from 3D T1-weighted MRI scans, we used FastSurfer network [23]. FastSurfer is a rapid and substantially verified deep learning pipeline that can analyze structural MRIs of the human brain in a completely automated fashion. FastSurfer Convolutional Neural Network (CNN) is composed of three fully convolutional neural networks that operate on coronal, axial, and sagittal 2D slice stacks, as well as a final view aggregation that combines the advantages of 3D patches and 2D slices. FastSurfer CNN was trained over 134 participant MRI scans aged 27-66 and was used to segment 96 distinct regional brain volumes. All MRI brain volumes were conformed to standard slice orientation and resolution (1 mm isotropic) before feeding to the different networks. Figure 1 shows output from CNN.

**Figure 2.**
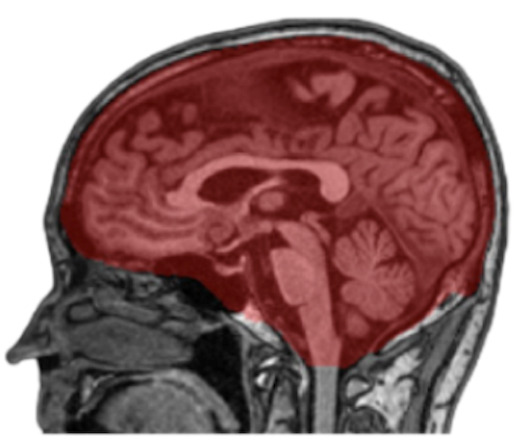
**Intracranial Volume Segmentation Results**. An example of intracranial volume (ICV) mask segmented by nnUNet model. Visualization overlay of the ICV segmentation in red was done on a raw sagittal T1 weighted MRI image using the MITK software tool.

### MRI Volumetric Measurement of Intracranial Volume (ICV)

To correct for differences in the head size of participants, a separate deep learning model was trained to segment ICV. To estimate ICV, we utilized 60 participants and annotated the intracranial compartment of these individuals manually according to Figure 2. We used these labeled data to train the nnUNet [22] for intracranial mask generation. As noted above, nnUNet is a self-configuring method for medical image segmentation. It includes preprocessing, network architecture, training, and post-processing for any new task. Figure 2 shows one such segmentation of ICV rendered using the MITK tool [24].

### Statistical Analyses

All statistical analyses were done in Python using sklearn and the scipy library [25]. Regression analyses were done adjusting for age and sex to understand the relationship between visceral fat volumes and brain regions. The type of brain regions evaluated from MR imaging whole brain parenchymal volumes: i) Total gray matter volume ii) Total white matter volume; Lobar volumes: i) Frontal ii) Temporal iii) Parietal iv) Occipital and early Alzheimer’s regions: i) Hippocampus ii) Posterior Cingulate gyrus iii) Precuneus. We repeated these analyses to evaluate for sex differences. As an exploratory analysis we also examined the relationship between subcutaneous fat and brain volumes. Binary logistic regressions adjusting for sex were then done to determine if the highest quartile of visceral fat predicted the lowest quartile of total gray and white matter volumes. Benjamini-Hochberg False Discovery Rate of 5% controlled for multiple comparisons [26].

**Table 1:** Participant Summary Information.

Category	Sex	n	Mean	Std Deviation	T-statistic	P-value
**Age**	All	10001	52.9 years	13.1 years		
**Visceral Fat**	Men	5284	3599.59 ml	2215.34 ml	-43.25	<.001
	Women	4717	1949.80 ml	1479.61 ml		
**Body Mass Index**	Men	5284	27.15	8.33	17.2	<.001
	Women	4717	25.22	6.55		
**Subcutaneous Fat**	Men	4420	1672.05 ml	716.42 ml	4.06	<.001
	Women	4034	1743.54 ml	898.60 ml		
**Fat Ratio**	Men	4420	2.1	1.08	-55.81	<.001
	Women	4034	1.07	0.55		
**Race and Ethnicity**						
**Caucasian**		5742				
**East Asian**		860				
**South Asian**		681				
**Indigenous**		11				
**Latin American**		178				
**West Asian, Arab,** **And North African**		255				
**Subsaharan African**		35				
**Mixed Race and Ethnicity**		1512				
**Not sure**		267				

## RESULTS

**Figure 3.**
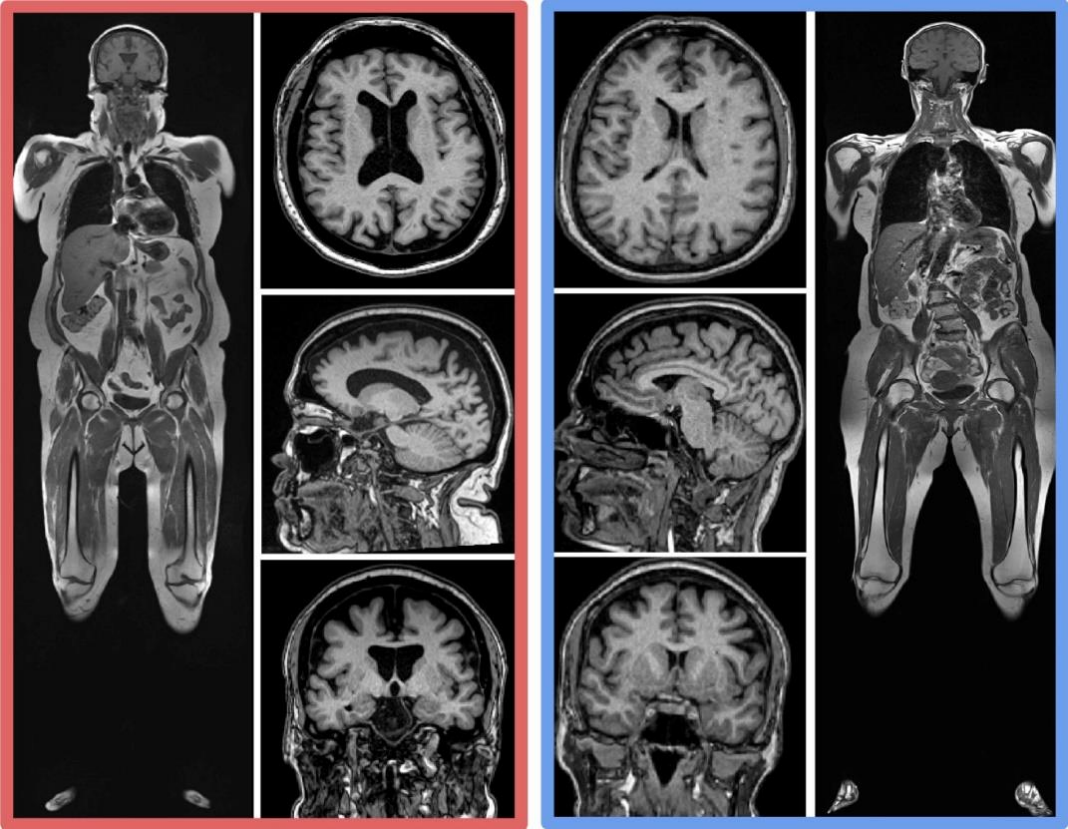
**Examples of Higher Body Fat and Brain Volume Loss**. This figure shows two examples of coronal T1 weighted whole body MRI in 61-year-old women, one with a high level of both vfat and sfat (red border) and the other with a normal amount of vfat and sfat (blue border). There is corresponding increased brain atrophy in the high vfat and sfat person as evidenced by enlarged ventricles and widened sulci on the T1 brain MRI images.

Figure 3 shows two examples of coronal T1 weighted whole body MRI in 61-year-old women, one with a high level of both vfat and sfat (red border) and the other with a normal amount of vfat and sfat (blue border). There is corresponding increased brain atrophy in the high vfat and sfat person as evidenced by enlarged ventricles and widened sulci on the T1 brain MRI images.

**Figure 4.**
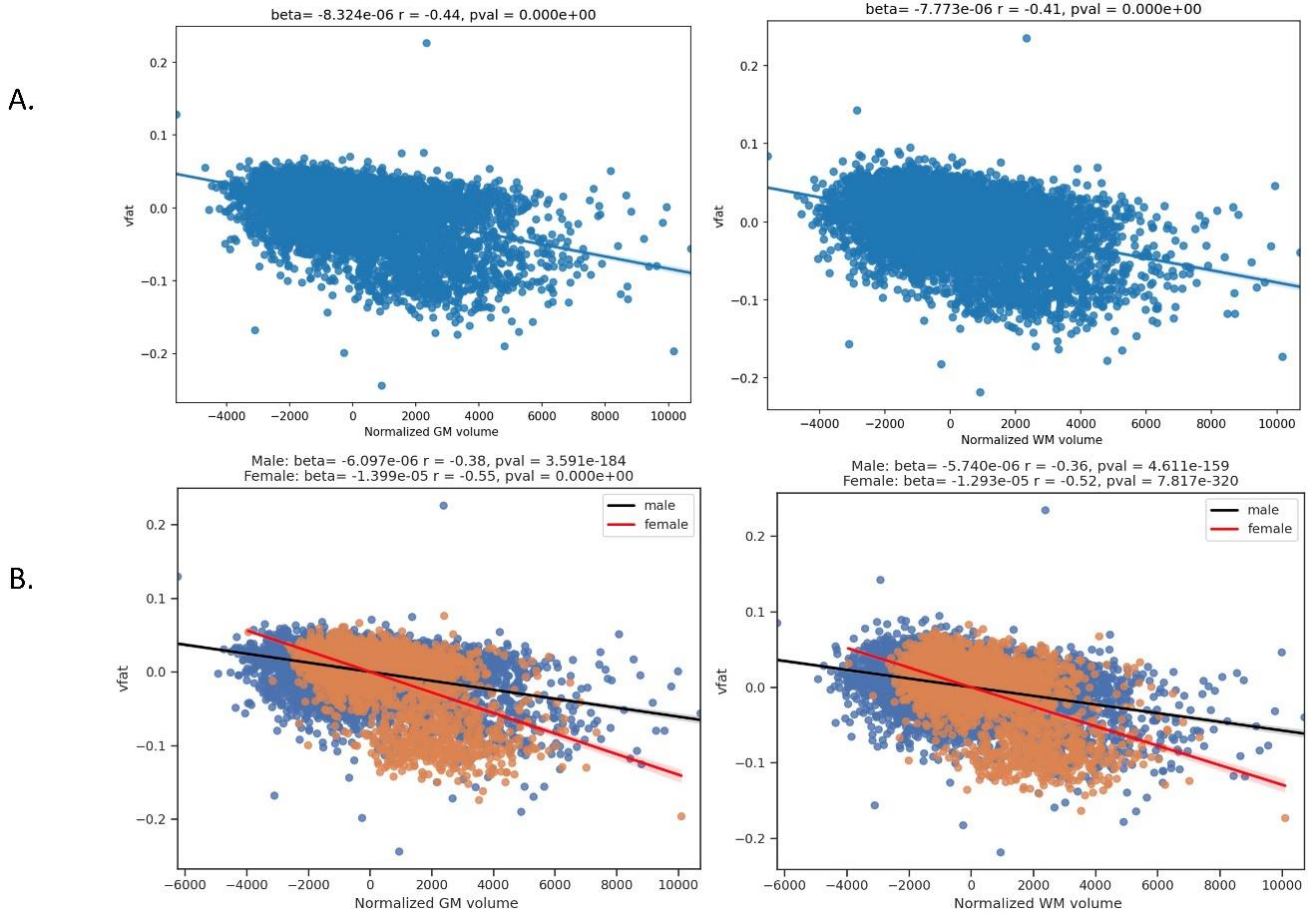
**Regression plots on the association of visceral fat and whole brain parenchyma tissue types**. This figure shows regression plots in which visceral fat is related to brain parenchymal volumes in part A: total gray matter and total white matter volumes normalized to total intracranial volume. Part A demonstrates that increased visceral fat is related to lower gray and white matter volumes in this sample of 10,001 individuals. Gray matter shows a higher magnitude of negative correlation than white matter to visceral fat. In Part B, sex differences are explored showing worse volume loss in women compared to men with increasing vfat in both total gray and white matter.

The Table 1 details participant summary data, drawn from U.S. and Canadian populations. The age range encompasses a large portion of the adult human lifespan, 18 to 90 years, with the average age being in midlife. Men had statistically significant higher visceral fat levels than women whereas subcutaneous fat levels were higher in women. Men on average also had higher BMI values than women. Mean visceral abdominal fat was 3417.81 ± 514.88 ml for overweight persons (BMI ≥ 25) and 5355.24 ± 505.04 for obese persons (BMI ≥ 30). Race and ethnicity data were obtained on 95.4% of the sample (n=9541) as the remainder of participants chose not to report this information. The remaining data is reported in Table 1. Additionally, our sample had 1023 persons with history of hypertension and 343 with type 2 diabetes mellitus with men more commonly affected than women (t=5.75, p=9.01e-09 and t=3.73 and p=.0002 respectively).

The main effect of age on gray matter and white matter is noted in Table 2, controlling for vfat and sex.

**Table 2:** Main Effect of Age on Total Gray and White Matter Volumes.

	n	r	CI95%		r2	adj_r2	p-value
**Gray Matter**	10001	-0.092	[-0.11,	-0.07]	0.008	0.008	4.02E-20
**White Matter**	10001	-0.097	[-0.12,	-0.08]	0.009	0.009	3.97E-22

The Figure 4 shows regression plots in which visceral fat is related to brain parenchymal volumes in part A: total gray matter and total white matter volumes normalized to total intracranial volume. Part A demonstrates that increased visceral fat is related to lower gray and white matter volumes in this sample of 10,001 individuals. Gray matter showed a higher magnitude of negative correlation than white matter to visceral fat. In Part B, sex differences are explored showing worse volume loss in women compared to men with increasing vfat in both total gray and white matter.

**Figure 5.**
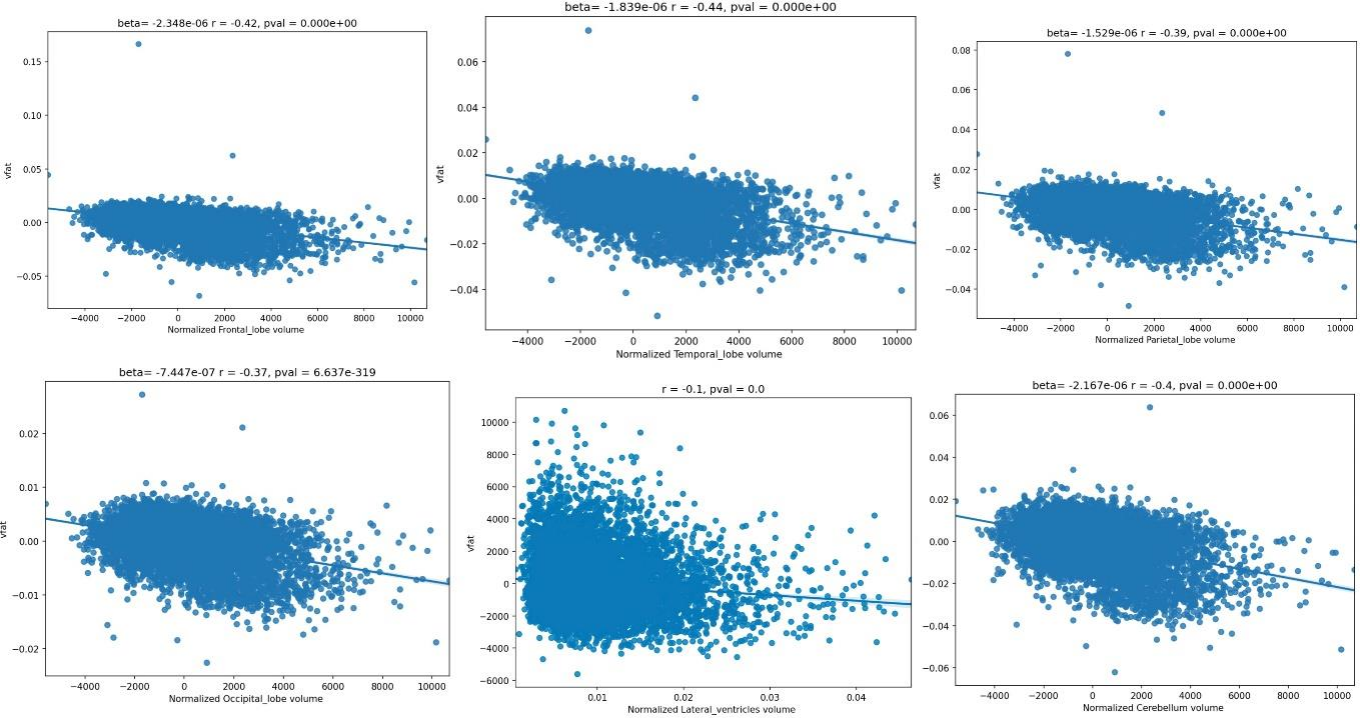
**Regression plots on the influence of visceral fat and brain lobar volumes**. These regression plots also show statistically significant relationships in which higher visceral fat is associated with lower lobar and cerebellar volumes. The strongest effect size is noted in the temporal lobe as evidenced by the largest magnitude of the r-value. The next largest r-values are noted in descending order in the frontal lobes, cerebellum, parietal lobe, and occipital lobe. A relatively smaller effect size was shown demonstrating smaller ventricular volume with higher vfat.

The Figure 5 regression plots also show statistically significant relationships in which higher visceral fat is associated with lower lobar and cerebellar volumes. The strongest effect size is noted in the temporal lobe as evidenced by the largest magnitude of the r-value. The next largest r-values are noted in descending order in the frontal lobes, cerebellum, parietal lobe, and occipital lobe. A relatively smaller effect size was shown demonstrating smaller ventricular volume with higher vfat.

**Figure 6.**
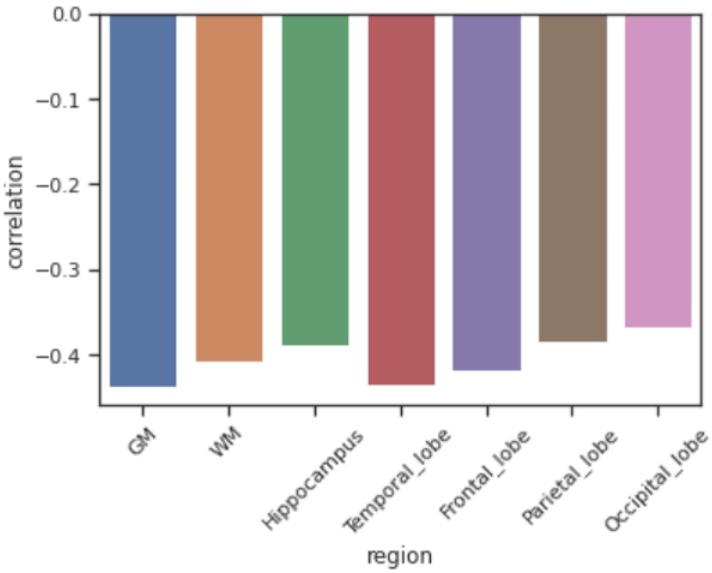
**Comparison of Partial Correlation Coefficients Between Visceral Fat and Representative Brain Region Volumes**. The results show that regional or lobar effects such as in the hippocampus and temporal lobes did not exceed the effect sizes in the whole brain parenchymal volumes, specifically total gray matter.

The Figure 6 shows comparisons of effect sizes between visceral fat several representative brain volumes under study. The results show that regional or lobar effects such as in the hippocampus and temporal lobes did not exceed the effect sizes in the whole brain parenchymal volumes, specifically total gray matter.

**Figure 7.**
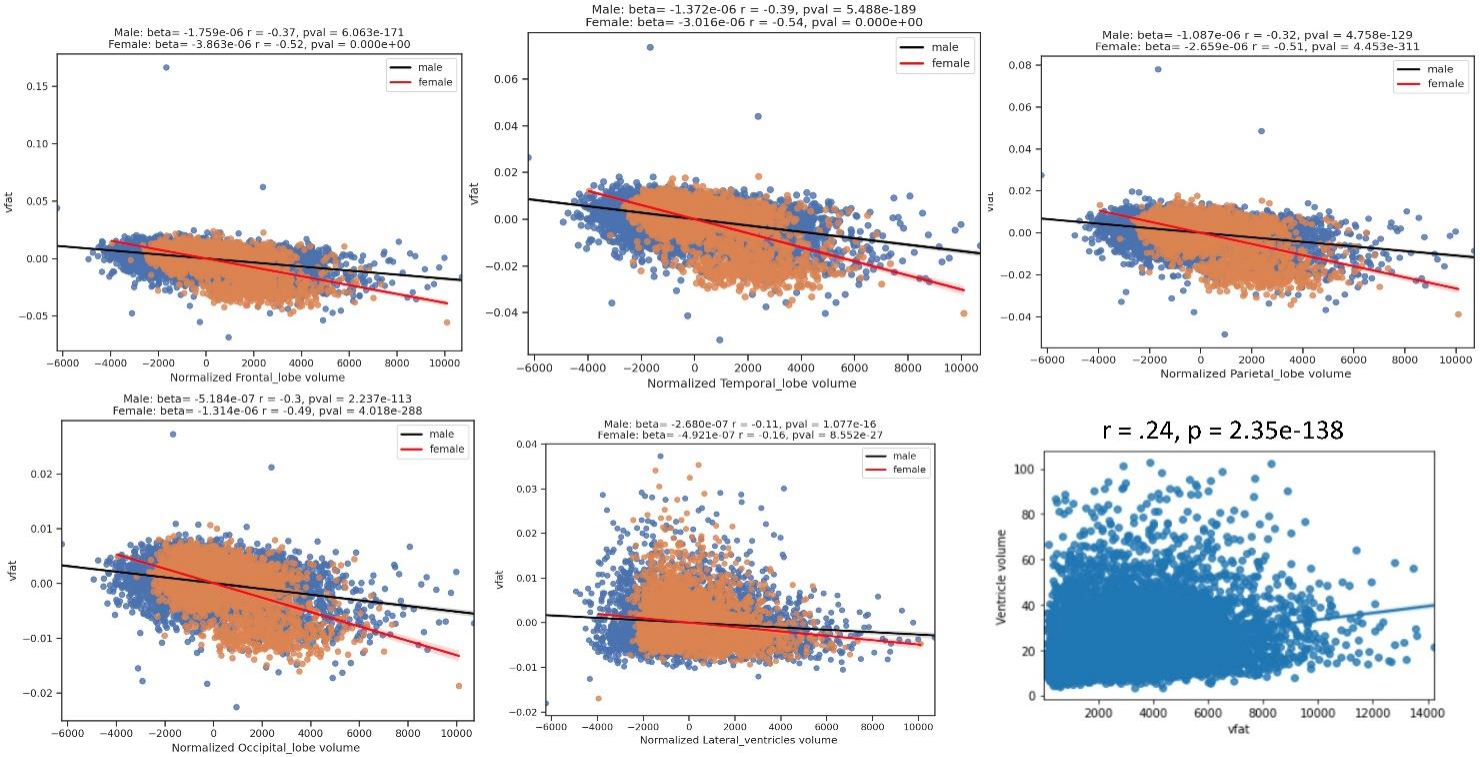
**Regression Plots of Visceral Fat with Normalized Brain Lobar Volumes in Men and Women and Non-Normalized Lateral Ventricular Volume**. The figure shows regression plots of visceral fat with normalized brain lobar volumes in men and women and non-normalized lateral ventricular volume. The results show that women experience lower volumes with higher vfat in women (red line) compared to men (black line) in the main lobar volumes but less so with normalized lateral ventricles. This figure also shows that in non-normalized lateral ventricles higher vfat related to increased ventricle size in a partial correlation plot adjusting for age.

The Figure 7 displays regression plots of visceral fat with normalized brain lobar volumes in men and women and non-normalized lateral ventricular volume. The results show that women experience lower volumes with higher vfat in women (red line) compared to men (black line) in the main lobar volumes but less so with normalized lateral ventricles. This figure also shows that in non-normalized lateral ventricles higher vfat related to increased ventricular size in a partial correlation plot adjusting for age.

**Figure 8.**
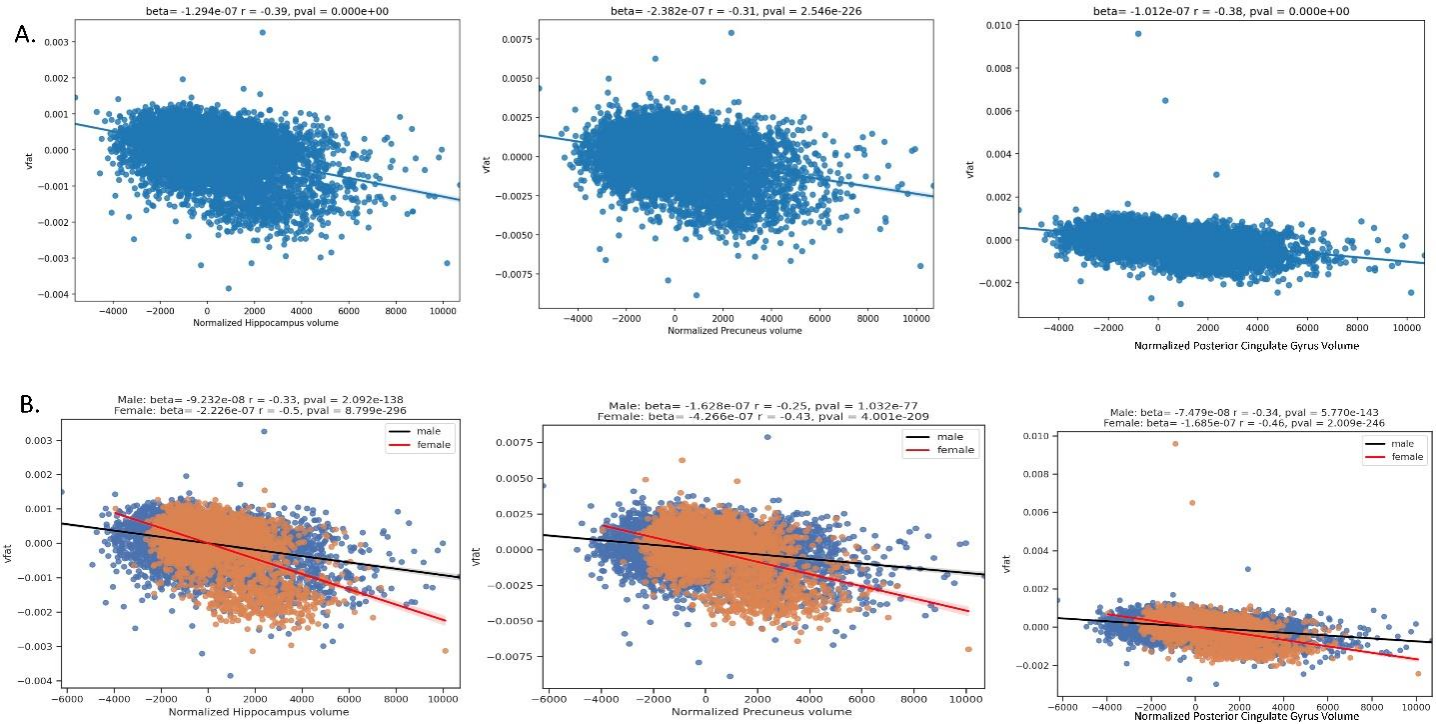
**Regression plots on the relationship of visceral fat and brain regions at risk for early AD pathology**. Part A shows the regression plots where higher vfat is related to lower normalized hippocampal, precuneus, and posterior cingulate gyrus volumes. Part B shows sex differences with the results in which women had a worse effect than men in these brain areas at risk for AD pathology.

The Figure 8 regression plots demonstrate the predictive value of higher visceral fat on lower volumes in brain specifically targeted by Alzheimer disease pathology: the hippocampus, posterior cingulate gyrus, and precuneus in Part A. The highest magnitude effect sizes are noted in the hippocampus, followed by the posterior cingulate and precuneus. Part B shows sex differences with the results in which women had a worse effect than men in these brain areas at risk for early AD pathology.

**Figure 9.**
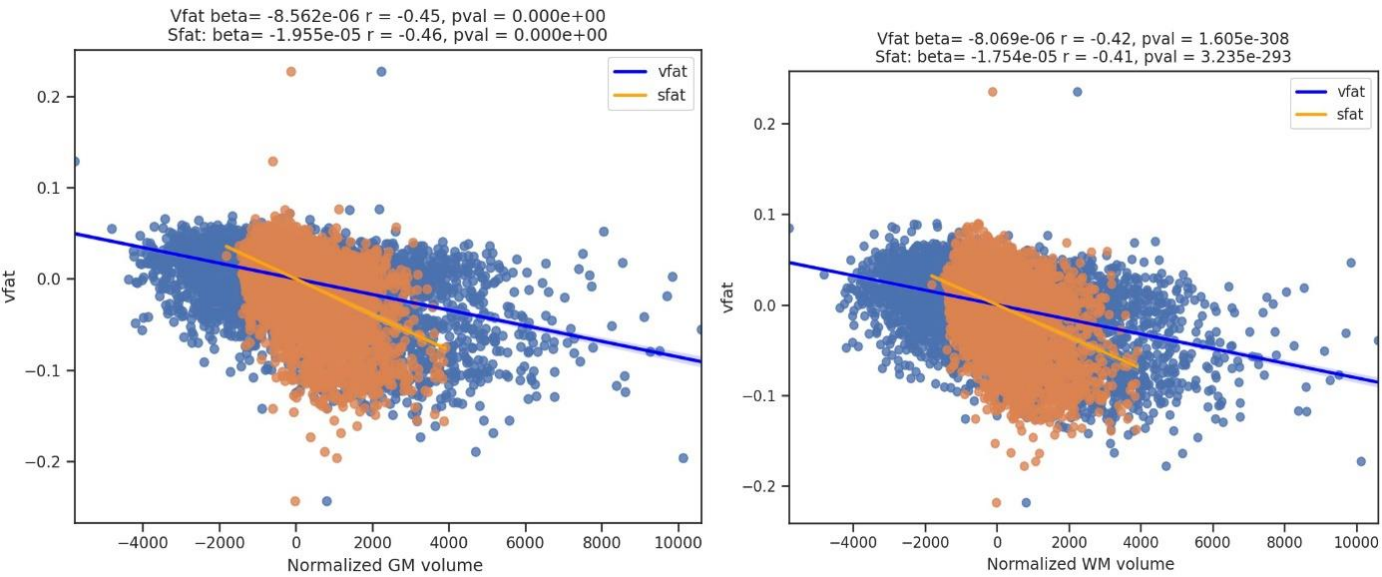
**Regression Plots of Visceral Fat and Subcutaneous Fat on Whole Brain Parenchymal Volumes**. This figure shows regression plots where the main effect of vfat (blue) is compared to sfat (orange) in normalized gray and white matter volumes, adjusting for age and sex. In both brain volumes, sfat showed a higher beta weight than vfat though the effect sizes were similar, beijing slightly higher with sfat.

The Figure 9 shows regression plots where the main effect of vfat (blue) is compared to sfat (orange) in normalized total gray and white matter volumes, adjusting for age and sex. In both brain volumes, sfat showed a higher beta weight than vfat though the effect sizes were similar though slightly higher with sfat.

**Figure 10.**
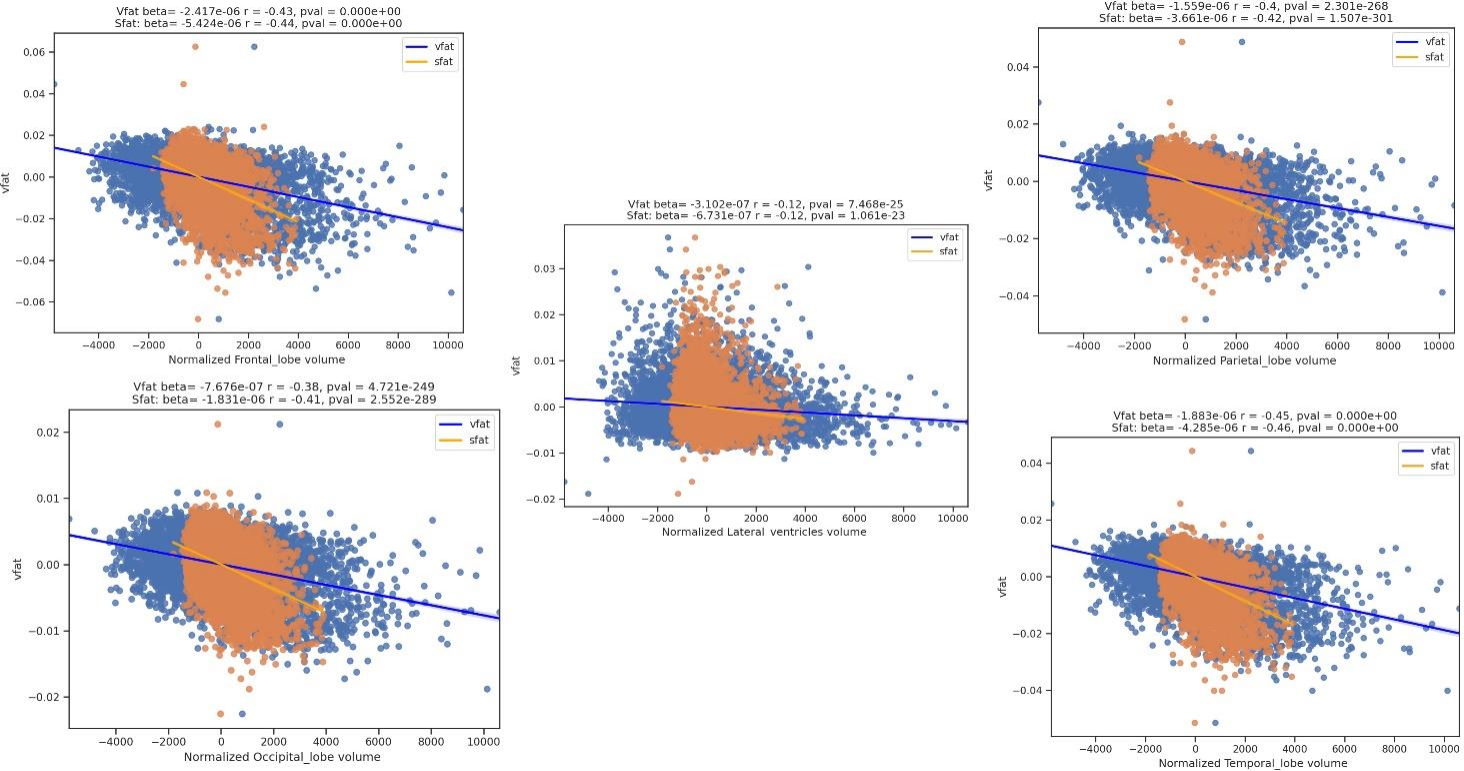
**Regression Plots of Visceral Fat, Subcutaneous Fat, Brain Lobar Volumes, and Lateral Ventricles**. This figure shows regression plots of vfat and sfat on brain lobar and ventricular volumes. As with gray matter and white matter volumes in Figure 9 there was mildly greater effect size between sfat and lower brain volumes than with vfat in these regions.

The Figure 10 shows regression plots of vfat and sfat on brain lobar and ventricular volumes. As with gray matter and white matter volumes in Figure 9 there was a mildly greater effect size between sfat and lower brain volumes than with vfat.

**Table 3:** Logistic Regression Results.

	p-value	std-err	beta coef	R2 adj	F-statistic	t-statistic
Gray Matter	<.001	472.337	-22956.288	0.191	2362.111	-48.602
White Matter	<.001	479.818	-21480.996	0.167	2004.269	-44.769
Hippocampus	<.001	27683.818	-1167845.265	0.151	1779.585	-42.185
Temporal lobe	<.001	2135.157	-103518.522	0.19	2350.588	-48.483
Frontal lobe	<.001	1617.543	-74453.991	0.175	2118.676	-46.029
Parietal lobe	<.001	2324.966	-97014.807	0.148	1741.177	-41.727
Occipital lobe	<.001	4598.304	-182152.936	0.136	1569.195	-39.613

**Figure 11.**
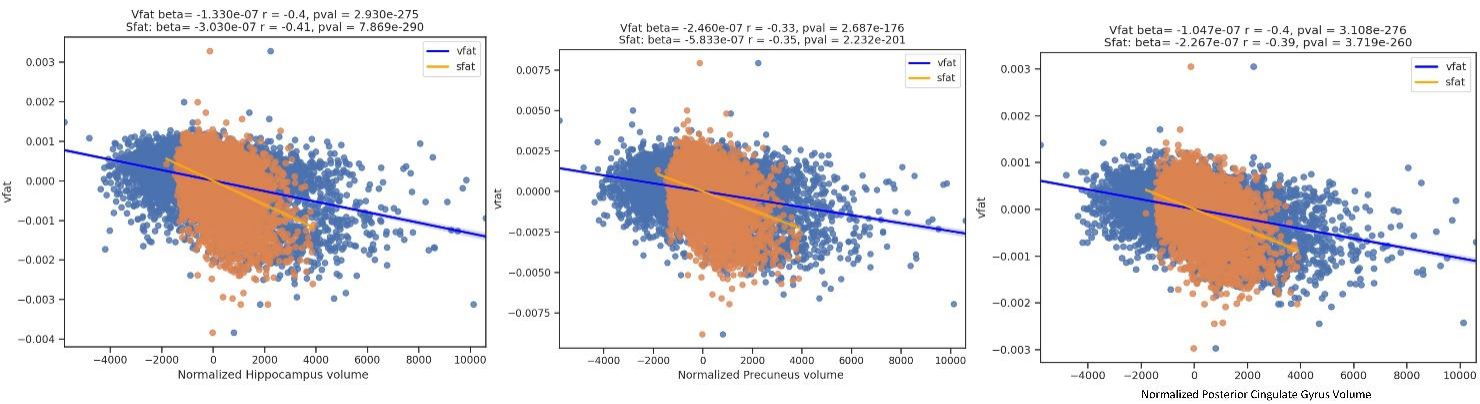
**Regression Plots of Visceral Fat and Subcutaneous Fat and Early Alzheimer Disease Regions**. This figure shows comparisons of vfat and sfat effects in the regions at risk for early AD pathology. Sfat was more tightly linked to lower volumes in these regions than vfat in terms of beta weights though effect sizes were similar.

The Figure 11 shows comparisons of vfat and sfat effects in the regions at risk for early AD pathology. Sfat was more tightly liked to lower volumes in these regions than vfat in terms of beta weights though effect sizes were similar.

With the logistic regression analyses, Visceral fat predicted increased risk for lower total gray matter (age 20-39: OR = 5.9; age 40-59, OR = 5.4; 60-80, OR = 5.1) and white matter atrophy: (age 20-39: OR = 3.78; age 40-59, OR = 4.4; 60-80, OR = 5.1). Additional statistical results related to this analysis are detailed in Table 3.

## DISCUSSION

This work demonstrated strong relationships in support of our hypothesis between higher visceral fat volume and visceral obesity and lower brain volumes from whole brain parenchyma to lobar volumes to specific areas relevant to AD pathology. Our work demonstrated sex differences on visceral fat relationships to brain volumes. We also found that subcutaneous fat had a stronger relationship to lower brain volumes than visceral fat. This relationship was not driven by the age of our sample given adjusting for age and sex in the regression models as well as our logistic regression results showing a higher burden of gray and white matter volume loss in younger participants.

Previous work has reported negative relationships between obesity and gray matter volume measures. A voxel based analysis in 1,428 Japanese participants demonstrated lower volumes with medial temporal lobes, anterior lobe of the cerebellum, occipital lobe, frontal lobe, precuneus, and midbrain with higher BMI [27], in partial agreement with our findings. Another study from the U.K. Biobank found that central obesity, as defined by combination of waist-hip-ratio, DEXA scans, and abdominal MRI correlated to lower gray matter volumes in 15,634 persons but did not detect any statistically significant correlations to white matter volume [28]. This partially replicated our findings in that we also found gray matter was more strongly related to higher visceral fat than white matter, yet we also detected atrophy in white matter related to visceral fat. Differences in our results could be related to the use of different scanners compared to U.K. Biobank.

A systematic review of 31 cross sectional diffusion tensor imaging studies showed a lower fractional anisotropy in the majority of those investigations related to increasing obesity [29]. An earlier study showed both lower fractional anisotropy and mean diffusivity as well as total white matter volume loss as shown on voxel based morphometry [30].

The influence of visceral fat on gray matter versus white matter has several potential explanations. First, to the extent that increased body fat is proinflammatory[31, 32], anti-inflammatory content of myelin [33] may render white matter relatively protected compared to gray matter. Additionally, gray matter shows greater reliance for protection from the blood brain barrier, with 3-5 times the capillary density as white matter [34]. Thus, the gray matter may show greater vulnerability to obesity related degradation of the blood brain barrier [35] and subsequently exposure to neuroinflammation[36]. Contrary to our expectations, higher visceral fat is actually related to lower lateral ventricular volumes, not larger as would be observed in aging [37]. However, the effect size is much weaker than our correlations and thus may not be a major effect particularly if the volume loss is of a relatively small magnitude. Ventricular enlargement is typically observed with a greater severity of atrophy, such as in Alzheimer’s [38]. We did show that non-normalized ventricular volumes did increase with visceral fat.

Among our lobar findings of lower volumes related to higher visceral fat, the temporal lobe was the most significantly influenced. This differential effect may in turn relate to the sensitivity of mesial temporal structures such as the hippocampus to atrophy in relation with higher body fat [39, 40]. At the same time, this process is not explained by amyloid and tau pathology as shown in a recent study of over 1,300 individuals [41]. Thus, the main mechanism by which there is increased vulnerability of these regions to visceral fat may be through inflammatory pathways. Concurrently, the posterior cingulate and precuneus - regions identified in our results and important for early AD pathology - also show higher vulnerability to peripheral inflammatory changes [42].

However, we also found that subcutaneous fat has a stronger relationship than visceral fat in terms of brain volume loss. One potential explanation is that both higher vfat and sfat have been shown to be related to increased levels of systemic inflammation, as shown in the Framingham study [43]. At the same time, sfat is up to 90% of today body fat whereas vfat is usually around 10% [44]. Thus, to the extent that both sfat and vfat are reservoirs of inflammation, we speculate that sfat may have a higher burden of inflammation given its higher volume of fat compared to vfat. This is suggested by literature results demonstrating that both vfat and sfat are related to lower brain volume [45]. A Korean study did show that vfat but not sfat was linked to lower cortical thickness but this lack of an sfat relationship could be due in part of a lower rate of obesity, and potentially sfat, in the Korean population compared to North America [46].

In our study, we demonstrated sex differences in the results in which women had a higher burden of brain atrophy with increased visceral fat than men. The challenge to placing the results in context is the lack of prior work specifically investigating visceral fat, brain volume loss, and sex differences. The largest study similar to ours in the U. K. Biobank lacked sex differences in total body fat and brain volume or microstructure [12]. A smaller study in late life obesity showed that men had greater impairment with white matter connectivity and higher BMI [47]. In another late life population with an age range of 70-84 years, women experienced a higher burden of temporal lobe volume loss with higher BMI and age [48]. The Women’s Health Initiative showed increased volume loss in women particularly in the hippocampus, frontal, and temporal white matter [49]. Differences in the ages evaluated as well as analysis techniques and lack of separate vfat and sfat quantifications and reliance on BMI may explain the differences in these results. Thus, future work on sex difference based on body fat will convey more information by evaluating anatomical specificity of body fat distribution.

Our study is not without limitations. Although we found statistically significant relationships between visceral fat levels and gray matter volume changes, their effect sizes were generally small [31]. Thus, the statistical significance of this work is influenced by the large sample size and less so by large effect size in any given set of regions. Also, the cross-sectional nature of this investigation precluded conclusions about causality that would have been possible with a longitudinal analysis. Additionally, we did not account for other lifestyle factors in this work such as physical activity, diet, and genetic variables and these data should be evaluated with respect to brain structure in future work. We also did not adjust for menopause, which has been related to visceral fat [50], in this investigation as we did not exclusively study our female sex participants as we have done so in prior work [51], though such questions are important for future work. Finally, while we did track atrophy with 3D T1 volumetric quantification, additional insights could have been obtained with advanced neuroimaging such as perfusion MRI and tractography.

Given the limitations, our study also has several strengths. Aside from having large sample size and including participants of various ages to enhance generalizability of findings. Furthermore, our U-Net architecture for image segmentation enabled accurate quantification of both visceral fat, subcutaneous fat and brain volumes amongst study population participants. We also explored sex difference as well in this work.

This research provides evidence of an association between high visceral fat and subcutaneous fat levels and reduced brain volumes, particularly those involved with cognitive function, suggesting visceral fat could play a part in cognitive decline and dementia risk through inflammation-related mechanisms. Further investigation should strive to better elucidate underlying mechanisms and discover possible interventions targeting abdominal fat reduction as a strategy to maintain brain health and cognitive functionality.
